# Low temperature Cu–Cu direct bonding in air ambient by ultrafast surface grain growth

**DOI:** 10.1098/rsos.240459

**Published:** 2024-09-11

**Authors:** Yun-Fong Lee, Yu-Chen Huang, Jui-Sheng Chang, Ting-Yi Cheng, Po-Yu Chen, Wei-Chieh Huang, Mei-Hsin Lo, Kuan-Lin Fu, Tse-Lin Lai, Po-Kai Chang, Zhong-Yen Yu, Cheng-Yi Liu

**Affiliations:** ^1^ Department of Chemical and Materials Engineering, National Central University, No. 300, Zhongda Road, Zhongli District, Taoyuan 32001, Taiwan

**Keywords:** near-atomic-scale surface, Cu–Cu direct bonding, electroplating, self-annealing, ultrafast grain growth

## Abstract

Fine-grain copper (Cu) films (grain size: 100.36 nm) with a near-atomic-scale surface (0.39 nm) were electroplated. Without advanced post-surface treatment, Cu–Cu direct bonding can be achieved with present-day fine-grain Cu films at 130℃ in air ambient with a minimum pressure of 1 MPa. The instantaneous growth rate on the first day is 164.29 nm d^−1^. Also, the average growth rate (∆*R*/∆*t*) is evaluated by the present experimental results: (i) 218.185 nm d^−1^ for the first-day period and (ii) 105.58 nm d^−1^ during the first 14-day period. Ultrafast grain growth and near-atomic-scale surface facilitate grain boundary motion across the bonding interface, which is the key to achieve Cu–Cu direct bonding at 130℃ in air ambient.

Chip-to-chip stacking is the key technology for 2.5-dimensional (2.5D) and three-dimensional (3D) integrated circuit packaging, for example, chip on wafer on substrate [1–3]. And, for chip stacking technology, Cu–Cu direct bonding is one of the most critical processes. Cu–Cu direct bonding has been demonstrated by various studies. In the past, many researchers passivated the Cu surface to improve the diffusion at the Cu–Cu bonding interface [4–7]. Also, the nanoscale Cu films made by sputtering can lower the bonding temperature [8]. Four key parameters for Cu–Cu direct bonding are the bonding temperature, pressure, ambient and surface roughness. Among them, it is very critical that the Cu–Cu direct bonding can be done at a low temperature (≤200℃), which would greatly save the thermal budget in the entire semiconductor process [9–11]. To achieve Cu–Cu direct bonding below 200℃, a relatively high bonding pressure has to be used such as 64–162 MPa [12–14]. The high external bonding pressure is the key to eliminate the voids resulting from the rough Cu surface at the bonding interface. However, the high external bonding pressure causes potential damage to integrated circuit chips. Hence, a combination of a low-roughness Cu surface (typically treated with chemical–mechanical polishing (CMP)) and controlled bonding ambient (vacuum, inert gas, or active acid bonding ambient) has been developed for Cu–Cu direct bonding below 200℃ with a relatively small bonding pressure [9–15]. The controlled bonding ambient is very challenging for Cu–Cu direct bonding in current semiconductor processes. Also, CMP is a very costly process, and the CMP process would bring in various impurities such as Si, SiC and Cl ions between the Cu–Cu bonding interface [16]. Thus, an untreated and low-roughness Cu surface for the Cu–Cu direct bonding process in air ambient would have a great advantage over the current reported Cu–Cu direct bonding processes. In this paper, a fine-grain Cu film was developed, which has a very low Cu surface roughness. With the near-atomic-scale Cu surface (without CMP surface treatment), Cu–Cu direct bonding can be done at low temperatures (≤200℃) in air atmosphere with a minimum bonding pressure of 1 MPa.

A Cu (500 nm)/Ti (200 nm) bi-layer was sputtered on Si substrates (1.5 cm × 1.5 cm), where Ti serves as the adhesion layer and Cu is the seed layer for the electroplating. Before the Cu plating process, the surface of the Cu seed layer was cleaned by the following two pretreatment processes. First, Cu (500 nm)/Ti (200 nm)/Si substrates were immersed in a micro-etching solution containing 0.36 M H_2_SO_4_ and 6.25 M sodium persulfate for 30 s. This step would micro-etch the Cu surface and remove possible oxides from the Cu surface. After the micro-etching process, the surface-cleaned Cu films (Cu (500 nm)/Ti (200 nm)/Si substrates) were immersed and stored in dilute acid solution with 0.09 M H_2_SO_4_. The purpose is to preserve the cleaned surface before Cu electroplating. After the surface cleaning processes, 5-μm-thick fine-grain Cu films were electroplated on the Ti (200 nm)/Cu (500 nm)/Si substrates. The Cu plating parameter and process flow are described as follows. A yttrium-oxide-coated Ti mesh is used as the anode. The current density and the plating time are 2 A dm^−2^ and 12 min, respectively. The Cu plating bath is composed of 1.5 M CuSO_4_, 0.18 M H_2_SO_4_ and 60 ppm chloride ion. To produce the fine-grain Cu film, two special additives were added to the Cu-plating solution, which are 300–500 ppm polypropylene glycol (PPG) and 7–9 ppm 3-methyl-2-cyclopentenone-4-ol. PPG additive functions as an inhibitor for reducing the Cu depositing rate and helping to form a fine-grain microstructure. 3-Methyl-2-cyclopentenone-4-ol is used as a leveller, which typically contains nitrogen heterocycles, sulfur heterocycles and thione groups. Levellers can suppress Cu deposition in certain irregular areas and reduce the surface roughness.

The as-plated Cu film was cross-sectioned using a focused ion beam (FIB, FEI Versa 3D) as shown in figure 1. The grain size was determined using the intercept procedure method (ASTM E112-13). In the FIB image of the fine-grain Cu film, 100 horizontal lines and 100 vertical lines were drawn, and the total number of Cu grains was counted along these lines. By taking the reciprocal of the total number of Cu grains per unit length, the average Cu grain size was determined to be 100.36 nm. The roughness (particularly below 5 nm) can be accurately obtained by integrating the shape function of the surface topology [17]. The shape function is a function of the roughness coefficient and Fresnel’s coefficient at the intended measured air–surface interface [17].

**Figure 1.**
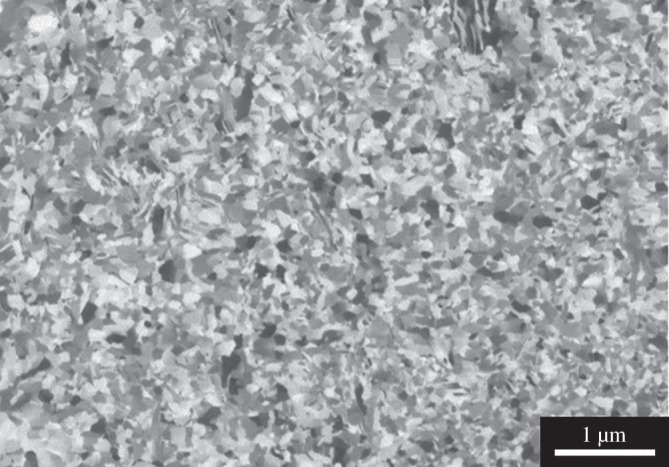
FIB cross-sectional image of the fine-grain Cu film.

In the present work, X-ray reflectivity (XRR) measurement was carried out on the surface of the fine-grain Cu film, which provides the reflection intensity and reflection coefficient at the air–Cu interface against the incident angle (2*θ*) of X-rays. With the reflection intensity versus 2*θ* relationship, as shown in figure 2, Fresnel’s coefficient at the air–Cu interface can be obtained through recursive algorithms [18–22]. Also, using Sinha’s approach, the roughness coefficient can be calculated by the reflection intensity and reflection coefficient obtained by XRR measurement versus the incident angle [18,20–23]. Note that the *R*^2^ value is 0.99. Knowing the roughness coefficient and Fresnel’s coefficient at the intended measured air–surface interface, the shape function can be obtained. Thus, the roughness (*R*_a_) of the as-electroplated fine-grain Cu films can be calculated by integrating the shape function, which is 0.942 ± 0.08 nm. Yet, the roughness obtained by XRR measurement for the Cu surface can be inaccurate due to noisy signals in modelling experimental data. Thus, atomic force microscopy (AFM) analysis has been done on the as-plated Cu surface in a 1 × 1 μm^2^ area. The surface roughness obtained from the AFM measurements is 0.39 nm, which has been included in table 2.

**Figure 2.**
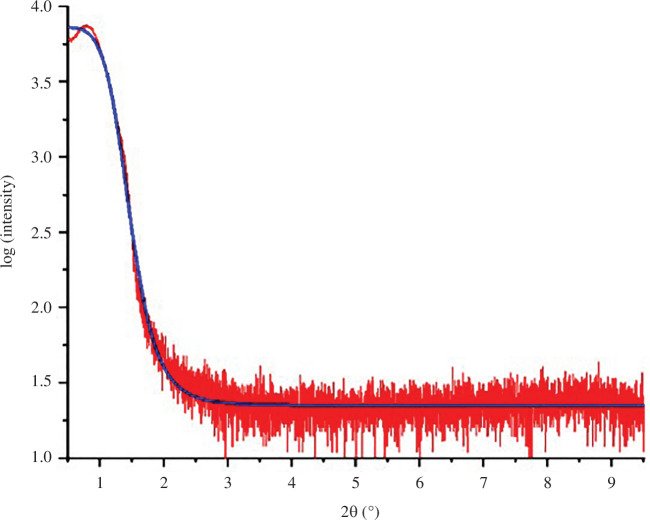
Mirror XRR measurement on the surface of the as-electroplated Cu film.

After the Cu plating process, the as-plated fine-grain Cu/Ti/Si substrates (1.5 cm × 1.5 cm) were cleaned immediately by dipping in a 0.5 wt% H_2_SO_4_ solution, rinsing with deionized water and blow-drying with N_2_ gas. Two surface-cleaned fine-grain Cu/Ti/Si samples were placed in a graphite bonding device to perform the Cu–Cu direct bonding process. A pressure of 1 MPa was applied to the Cu–Cu bonding samples. The bonding temperatures are set at 450, 350, 300, 200, 150 and 130℃ for 30 min. The bonding atmosphere is air ambient.

After the Cu–Cu direct bonding process, the Cu–Cu bonding samples were cross-sectioned and examined by FIB, as shown in figure 3. Remarkably, as depicted in figure 3*f*, Cu–Cu direct bonding can occur at temperatures as low as 130℃ in air ambient. No observable micro-voids are present at the bonding interface, as indicated by the white arrows in figure 3*f*, demonstrating a continuous bonding interface. The slightly distorted bonding interface in the bottom image is due to chipping and missing of the left-corner Si during the polishing process. For the higher bonding temperatures at 450, 350 and 300℃, as seen in figure 3*a–c*, the Cu grain boundary migration across the bonding interface can clearly be observed. It indicates that much faster grain growth would occur at the initial bonding interface at higher bonding temperatures. Electron backscatter diffraction (EBSD) analysis has been done on the cross-sectioned Cu–Cu bonding interfaces. The EBSD scanning images are shown in figure 4*a,b*. EBSD images can reveal the bonding quality, which depends on the diffusion across the bonding interface. As seen in figure 4*a*, the bonding interface for the 130℃ sample is relatively straight. Compared with the bonding interface at 130℃, we can observe that the curvature shows the bonding interface at 200℃ (figure 4*d*), as indicated by a dashed black ellipse. From the EBSD images, we can also observe that the grain size increases with the bonding temperature and that an annealing twin boundary forms. We also performed shear tests with a shear test tool (Dage 4000PXY) to evaluate the mechanical strength of the Cu–Cu bonding. The shear strength of the Cu–Cu bonding samples with different bonding temperatures is quite similar, ranging from 20.24 to 22.02 MPa. Also, note that the Cu–Cu bonding sample at 130℃ has the least shear strength of 20.24 MPa. And, interestingly, the highest shear strength (22.02 MPa) occurs for the 200℃ Cu–Cu bonding sample.

**Figure 3.**
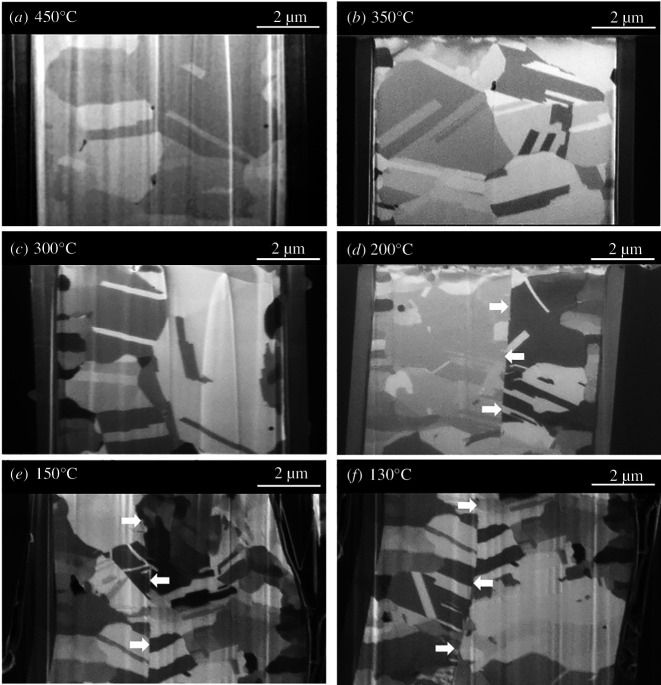
FIB images of Cu–Cu direct bonding samples at (*a*) 450°C, (*b*) 350°C, (*c*) 300°C, (*d*) 200°C, (*e*) 150°C and (*f*) 130°C.

**Figure 4.**
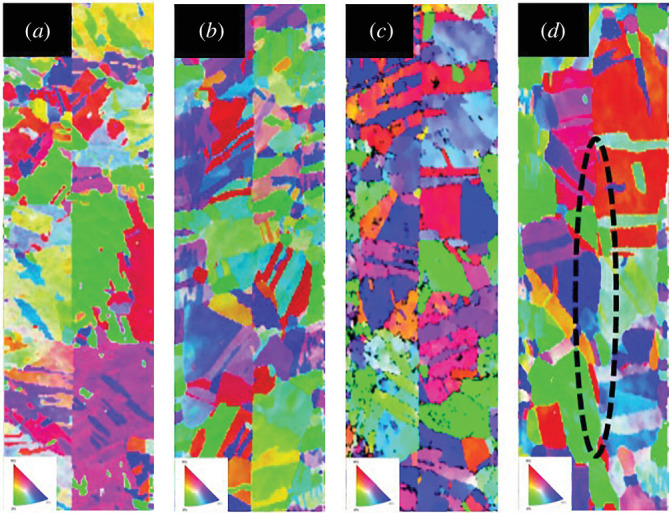
EBSD layered images of Cu–Cu direct bonding samples at (*a*) 130°C, (*b*) 150°C, (*c*) 200°C and (*d*) 300°C.

The bonding samples under three low bonding temperatures (130, 150 and 200℃) are more critical to demonstrate the bonding quality for this work. Thus, the *I*–*V* behaviour of the Cu–Cu bonding samples (130, 150 and 200℃) was measured with a probe station and Keithley 2400 measuring unit. Two electrical probes were placed on the centre point of two Cu-bonded layers and separated by about 1 mm. *I*–*V* curves of all three bonding samples (130, 150 and 200℃) demonstrate ohmic behaviour, i.e. linear curves in *I*–*V* plots. The slope of the regression lines in the measured *I*–*V* plot represents the reciprocal of the sheet resistance (*R*_s_). And, the resistivity (*ρ*) can be calculated by the expression 4.532(*R*_s_)(*L*), where *L* is the measurement length (about 1 mm) between two electrical probes. Table 1 shows the slope of the *I*–*V* curve and the calculated resistivity (*ρ*) of the Cu–Cu bonding samples under three low bonding temperatures (130, 150 and 200℃). The measured resistivity of Cu–Cu bonding samples with different bonding temperatures is of the same order as the resistivity of pure Cu (1.7 × 10^–8^ Ω m).

**Table 1.** The slope of *I*–*V* curve, and the calculated resistivity (*ρ*) of the Cu–Cu bonding samples under three low bonding temperatures (130, 150 and 200℃).

temperature (℃)	200	150	130
slope of *I*–*V* curve	0.1613	0.1094	0.1181
*ρ* (10^−8^ Ω m)	2.81	4.14	3.84

Cu–Cu direct bonding has been demonstrated by numerous studies. All Cu–Cu direct bonding done below 200℃ is summarized in table 2, which is categorized with four key parameters, i.e. bonding temperature, pressure, ambient and surface roughness. The majority of Cu–Cu direct bonding below 200℃ has to be done with CMP surface treatment [3,12,15,24]. The typical surface roughness of the CMP Cu surface is between 1.2 and 6.5 nm [12,15,17,24]. Most Cu–Cu direct bonding without CMP surface treatment has to be done at a bonding temperature of over 200℃. One exception is that the Cu–Cu direct bonding can be achieved below 200℃; however, it requires a vacuum bonding ambient [10]. The present work significantly demonstrates that Cu–Cu direct bonding can be achieved at 130℃ in air ambient.

**Table 2.** Summary of present and previous Cu–Cu direct bonding results and parameters.

bonding condition	temperature (℃)	pressure (MPa)	time (min)	surface roughness (nm)	bonding ambient	surface treatment
current work	130	1	30	0.94 (0.39 nm, AFM)	air	no CMP
[10]	150	0.876	60	6.5	vacuum	no CMP
[10]	200	0.876	30	6.5	vacuum	no CMP
[14]	146	64	20	5	nitrogen gas	CMP
[12]	150	162	5	1.82	nitrogen gas	CMP
[13]	150	0.69	30	6.5	vacuum	CMP
[13]	200	0.69	30	6.5	vacuum	CMP

Figure 5*a–d* shows the FIB cross-sectional images on the fine-grain Cu films stored at room temperature for 1, 5, 7 and 14 days. The self-annealing in the fine-grain Cu films can be clearly observed. With the intercept procedure method, the average grain size of the fine-grain Cu films stored at room temperature for 1, 5, 7 and 14 days was calculated to be 537.73, 787.42, 1265.89 and 1578.52 nm, respectively.

**Figure 5.**
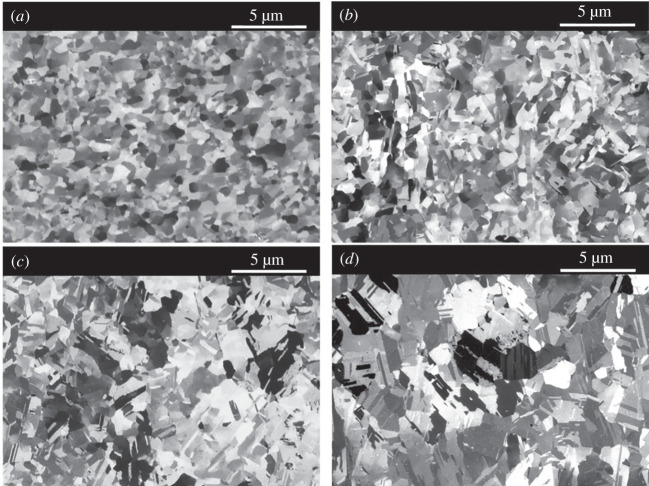
FIB images of the fine-grain Cu films with different self-annealing times: (*a*) 1 day, (*b*) 5 days, (*c*) 7 days and (*d*) 14 days.

The average grain size of the fine-grain Cu films is plotted against the storage time in figure 6*a*. In figure 6, results of previous research on the self-annealing behaviour of Cu films also included [25–27]. The curves in figure 6*b* were fitted to the grain size data in figure 6*a* with the Ostwald ripening model, which is typically written as equation (1.1):

(1.1) $$\frac{dR}{dt}=C_{1}\gamma \kappa =C_{2}\left(\frac{1}{R}\right),$$

**Figure 6.**
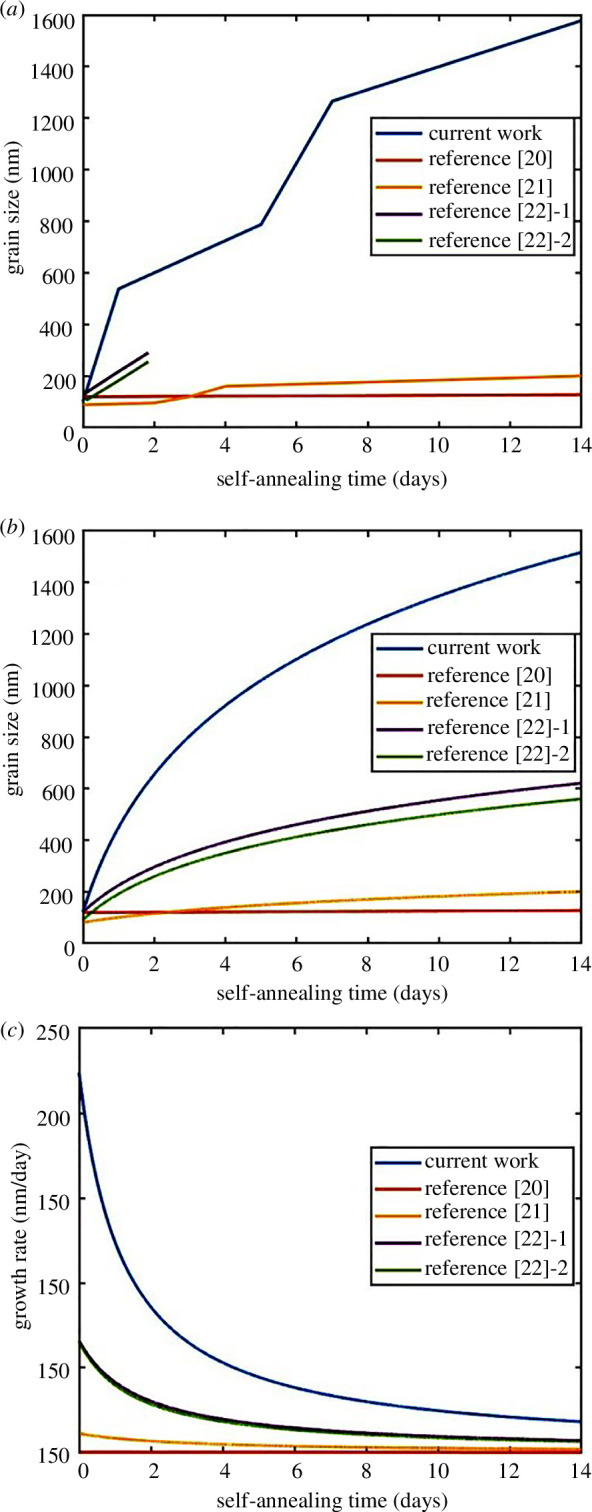
Grain size and growth rate of fine-grain Cu films with different self-annealing times. (*a*) Grain size. (*b*) Fitted curves of grain size. (*c*) Fitted curves of grain growth rate.

where *C*_1_ and *C*_2_ are constants, *γ* is the grain boundary energy, *κ* is the grain curvature and *R* is the average radius of Cu grains. The product of *γ* and *κ* is the net driving force for Cu grain growth. Assuming that the average radius of Cu grains (*R*) is an exponential function of time (*t*), which can be expressed as *C*_3_(*t* + *a*)^*n*^, where *a* and *C*_3_ are constants, then equation (1.1) can be rewritten as equation (1.2), where *b* is the integrated constant:


(1.2) $$\frac{dR}{dt}=b\left(\frac{1}{(t+a{)}^{n}}\right).$$

By integrating both sides of equation (1.2) with *t*, equations (1.3) and (1.4) can be derived under two conditions for the value of *n*:

(1.3) R=b⋅ln⁡(t+a)(for n=1),

(1.4) $$R=nb\frac{1}{(t+a{)}^{n-1}}\;(\text{for }n>1).$$

By fitting the data curves in figure 6*a* with equations (1.3) and (1.4), the constants of *a* and *b* in those equations can be obtained. Also, we found that the *R*^2^ value (with regression analysis) is over 0.95, as *n* equals 1. Thus, the constants of *a* and *b* fitted by equation (1.3) were chosen for equation (1.2). The curves of grain growth can be plotted, as shown in figure 6. With the curves in figure 6*c*, the instantaneous growth rate can be defined at any particular self-annealing time. Thus, we can estimate the instantaneous growth rate on the first day as 164.29 nm d^−1^. Also, the average growth rate (∆*R*/∆*t*) is evaluated by figure 5*a* and found to be as follows: (i) 218.185 nm d^−1^ for the first-day period and (ii) 105.58 nm d^−1^ during the first 14-day period. Comparing the present work with previous results, as shown in figure 6*c*, an ultrafast self-annealing grain growth behaviour is found in the present fine-grain Cu films. Hence, we believe that the ultrafast grain growth facilitates the motion of the grain boundary across the bonding interface, which should be the key to achieve Cu–Cu direct bonding at the low bonding temperature of 130℃ in air ambient. A similar growth model of fine-grain Cu produced through severe plastic deformation has been reported [28]. The ultrafast diffusion was proposed to be the main mechanism for the grain growth model of fine-grain Cu [29].

In conclusion, the current work demonstrates that without any advanced post-electroplating surface treatment, Cu–Cu direct bonding can be achieved at 130℃ in air ambient with a minimum bonding pressure of 1 MPa. The bonding interface contains no micro-voids. The achievement of the present Cu–Cu direct bonding is attributed to two key features of the Cu electroplated films, which are the near-atomic-scale Cu surface and ultrafast self-annealing grain growth. The low Cu surface roughness eliminates potential micro-voids forming at the bonding interface. And then, the ultrafast grain growth facilitates the motion of the grain boundary across the bonding interface, which should be the key to achieve Cu–Cu direct bonding at the low bonding temperature of 130℃ in air ambient. By the Ostwald ripening model, the instantaneous growth rate (d*R*/d*t*) of the present fine-grain Cu films is calculated to be 164.29 nm d^−1^ on the first day.

## Data Availability

We provide the experimental data for all of our experiments in the paper. The data that support the findings of this study are available on request from the corresponding author, C.-Y.L. The data are not publicly available due to restrictions, e.g. information that could compromise the privacy of research participants. Supplementary material is available online [30].
